# Acute Appendicitis in the Elderly: A Literature Review on an Increasingly Frequent Surgical Problem

**DOI:** 10.3390/geriatrics6030093

**Published:** 2021-09-18

**Authors:** Sintija Lapsa, Arturs Ozolins, Ilze Strumfa, Janis Gardovskis

**Affiliations:** 1 Department of Surgery, Pauls Stradins Clinical University Hospital, 13 Pilsonu Street, LV-1002 Riga, Latvia; arturs.ozolins@stradini.lv (A.O.); janis.gardovskis@stradini.lv (J.G.); 2 Department of Surgery, Riga Stradins University, 16 Dzirciema Street, LV-1007 Riga, Latvia; 3 Department of Pathology, Riga Stradins University, 16 Dzirciema Street, LV-1007 Riga, Latvia; ilze.strumfa@rsu.lv

**Keywords:** elderly, acute appendicitis, surgery

## Abstract

With increased life expectancy and the growing total population of elderly patients, there has been rise in the number of cases of acute appendicitis in elderly people. Although acute appendicitis is not the most typical pathological condition in the elderly, it is not uncommon. Most of these patients require surgical treatment, and as with any acute surgical pathology in advanced age, treatment possibilities are affected by comorbidities, overall health status, and an increased risk of complications. In this literature review we discuss differences in acute appendicitis in the elderly population, with a focus on clinical signs, diagnostics, pathogenesis, treatment, and results.

## 1. Introduction

Acute appendicitis is one of the most common surgical pathological conditions, with a lifetime risk of 7–8% [1]. In the Western world the incidence of acute appendicitis has been stable over the last 20 years after a decrease in the 20th century, but recently there has been an increase in incidence in South America, Asia, and the Middle East [2]. The incidence of acute appendicitis in the elderly is rising due to a longer life expectancy [3]. Although there is a reduction in the incidence of acute appendicitis after adolescence, acute appendicitis in the elderly is not uncommon—15% of patients above the age of 50 that present in the emergency department with acute abdominal pain have acute appendicitis, and it is the second most common acute surgical pathological condition [4], with an increasing frequency [5].

The definition of the term “elderly” is somewhat unclear. Conventionally, it has been considered as a chronological age of 65 or older, but the World Health Organization and the Japanese Geriatrics Society have recently suggested a cut-off value 75 or older based on improvements in physical function over the last 10–20 years [6]. There is an ongoing discussion regarding how the term “elderly” should reflect chronological and biological age, independence level, and health status [7], et cetera. The age for the elderly in the available studies ranges from 60 to 80 years [5,8,9,10,11,12]. Therefore, in this text the term “elderly” will be used in accordance with the reviewed studies.

## 2. Clinical Signs

The most common symptoms associated with acute appendicitis are also observed in elderly patients—lower abdominal pain (93.9–97.6%), anorexia (57.6–67.0%), nausea and vomiting (45.5–68.3%), shifting pain (30.3–45.1%), right iliac fossa pain (60.6%), and pyrexia (21.2–26.8%) [13,14]. Elderly patients may not have conclusive clinical signs of acute appendicitis, but signs of peritonitis—abdominal distention, reduced abdominal wall movement, severe tenderness, localized and generalized guarding—are more pronounced [4].

With increasing age, the ability to sense pain is decreased. Data from a study on abdominal pain perception in the elderly suggest a loss of spinal afferent innervation in humans [15]. Based on studies in mice, there could also be decreased ability to transduce inflammatory mediators [15].

Due to the lower basal temperature, diminished thermoregulatory response, and abnormalities in the production of and response to endogenous pyrogens, approximately 20–30% of the elderly population with acute infection will have a lower or absent fever response [16].

## 3. Diagnostics

### 3.1. Laboratory Values

There are several studies on different diagnostic and/or predictive laboratory values (for example, complete blood count, white blood cell count, neutrophil-to-lymphocyte ratio, mean platelet volume, and total bilirubin levels [17,18,19]) to differentiate acute appendicitis or predict perforation of the appendix. Only a few studies have been done in the geriatric population and these yielded different objectives and results. Elevated white blood cell (WBC) count, neutrophil-to-lymphocyte ratio, platelet-to-lymphocyte ratio, and delta neutrophil index (DNI) are possible markers of perforated appendicitis in the elderly, with DNI being the only one that can significantly predict perforation, with a cut-off value of 1.4% (sensitivity 67.7%, specificity 90.0%) [10]. While comparing geriatric versus non-geriatric patients, WBC, lymphocyte count and neutrophil/WBC ratio were statistically higher in the non-geriatric group, with only WBC being statistically significant [20].

### 3.2. Scoring Systems

Different scoring systems for acute appendicitis (Alvarado score, RIPASA, Lintula score: see Table 1) have been established for use in the younger population but lack evidence or usefulness in elderly patients. All of these scoring systems are based on the same principles. They include symptoms (nausea and vomiting, pain in the right iliac fossa (RIF), migration of pain), clinical signs (rebound tenderness, body temperature), and laboratory values (leukocytosis, C-reactive protein (CRP)) but have different interpretations of these findings and different score values. There are few small retrospective studies assessing these scores in the elderly population, predominantly using the more popular Alvarado score. Modifying the Alvarado score results to five or greater may lead to a timely diagnosis [14], but this does not help to differentiate complicated versus uncomplicated appendicitis [21]. In a single study, after score modification (excluding nausea and bowel sounds) the area under curve (AUC) in the ROC analysis reached 97.5% (95% CI: 95.0–100.0%) and 95.1% (95% CI: 90.5–99.6%) for the Alvarado and Lintula scores, respectively, as compared with Alvarado and Lintula scores without modification of 96.9% (CI: 94.0–99.8%) and 92.8% (CI: 87.4–98.2%), respectively [22]. Different cut-off points for these scores were thus created for adult and pediatric populations. Although there are no widely accepted and/or validated scoring systems in the elderly, a recent prospective study showed the high value of the Diagnostic Score—a calculated score that includes clinical history and clinical signs. The Diagnostic Score shows at least equal sensitivity and specificity for the elderly as compared to younger patients. The sensitivity in the elderly is 90% (CI: 80–97%) vs. 90% (CI: 85–95%) in younger females and 93% (CI: 88–96%) in younger males, while the specificity in the elderly is 95% (CI: 89–99%) vs. 85% (CI: 74–94%) in younger females and 84% (CI: 74–92%) in younger males [23].

### 3.3. Imaging

Abdominal ultrasound (US) is the first-line imaging choice in different populations (children, pregnant women). However, the overall sensitivity of 86% and specificity of 81% of US limit its usefulness in older populations [27].

Acute appendicitis is not the most common pathological condition in elderly patients with acute abdominal pain, as it presents in 3% [28] to 5% [29] of all patients requiring computed tomography (CT). However, liberal use of CT is suggested in elderly patients with acute abdominal pain due to broad spectrum of conditions, as it can influence the treatment plan in up to 65% of patients with positive CT findings, medical management in 52%, and surgical management in 48% [28]. Unenhanced CT has been suggested for triaging elderly patients with acute abdominal pain [29]. There are no data on sensitivity and specificity exclusively in the elderly, but in a meta-analysis in adult populations, the results of second-line US (sensitivity 83.9%, specificity 90.9%), CT (sensitivity 89.9%, specificity 93.6%), and MRI (sensitivity 89.9%, specificity 93.6%) are comparable and accurate [30].

Most elderly patients undergo CT scan preoperatively (74.6–97.9% [14,31]).

## 4. Uncomplicated versus Complicated Appendicitis

With the presumption that the complicated and uncomplicated forms of appendicitis are two discrete entities with different pathophysiology [32] came a shift in the management of acute appendicitis at the beginning of the 2010s. It has been proposed that there are different forms of immunopathogenesis [33], even with a genetically driven inflammatory response [34].

Complicated appendicitis is characterized as gangrenous with transmural necrosis, perforated, or with the presence of an appendicolith, periappendicular/abdominal abscess, and/or diffuse peritonitis. Uncomplicated appendicitis features suppurative/phlegmonous changes only [35].

Discriminating uncomplicated versus complicated appendicitis before surgery is challenging. The CT scan is one of the most precise methods. In a recent meta-analysis, 14 signs of complicated appendicitis were proposed, with several of them having higher specificity (>70%)—extraluminal appendicolith, abscess, extraluminal air, appendiceal wall enhancement defect, ileus, periappendiceal fluid collection, ascites, intraluminal air, and intraluminal appendicolith [36].

There have been two attempts to propose a scoring system in adults based on clinical and imaging (either US or CT) signs to differentiate between the two entities [37,38], but neither of these has been validated in prospective studies. Both proposed scoring systems included age as a predictive sign, either above 45 or 52 years of age.

The prevalence of complicated appendicitis increases with age: 13.6–20.97% in patients younger than 40 [5,11,39], 37.5% in those aged 40–64 years [39], 43.97% in those aged 65–74 [39], and 56.84–63.0% in those aged above 75 [5,39], with an even bigger increase after age of 80 (64.9–72.7%) [12,13].

There are few studies in the elderly population differentiating complicated versus uncomplicated appendicitis based on clinical signs. A period of time over 24 h from onset of symptoms to emergency department arrival, heart rate >90 beats/minute, respiratory rate >20 breaths/minute, and generalized abdominal guarding could be predictive signs of complicated appendicitis in the elderly [40].

## 5. Treatment

### 5.1. Conservative Treatment

In a recent large randomized study comparing antibiotics with appendectomy (including patients with appendicolith), conservative treatment was proven non-inferior to appendectomy. After 90 days, appendectomy was performed in 29% of patients in the conservatively treated group (25% without appendicolith, 41% with appendicolith) [41]. These data correspond to a long-term (5 years) follow-up of recurrence of appendicitis of 39.1% [42]. In some of the recent meta-analyses from Germany and China, appendectomy still appears more effective as the definitive treatment, with different results regarding complications—fewer complications in conservative treatment were found in a Chinese meta-analysis and no significant difference was reported in a German publication [43,44].

A small study of nonoperative treatment in the elderly (>80) showed a 20% recurrence rate, suggesting that antibiotics could be a treatment option in selected elderly individuals with uncomplicated appendicitis [45].

### 5.2. Surgical Treatment

Diagnostic challenges in the elderly are reflected in the time spent from admission until surgery—67.2–71.4% of elderly patients are operated on more than 12 h after admission compared with 34.4% of younger patients [31]. In addition, 37% of elderly patients are operated on more than 24 h after admission, although without any impact on postoperative complications [46]. Increased use of imaging results in lower rates of negative appendectomies in the elderly at 9–10%, while in younger patients the frequency of negative appendectomies reaches 15.7% [31].

The laparoscopic appendectomy was first described by Semm in 1983 [47]. Since then, the laparoscopic approach has become the standard of care in acute appendicitis in the adult population. When choosing the appropriate surgical method, physiological effects of pneumoperitoneum should be taken into consideration—decreased pulmonary compliance, urinary output, glomerular filtration rate, and renal blood flow, and increased heart rate, mean arterial pressure, and cardiac output [48].

The advantages of the laparoscopic appendectomy over open surgery in the general population have been proven and are well known—decreased frequency of postoperative wound infection, lesser postoperative pain, and shorter length of hospital stay. However, there is an increased incidence of intraabdominal abscess formation. Although few data are available on the elderly population, in a meta-analysis from 2012 of the elderly population (>60 years old), postoperative mortality and complications as well as length of hospital stay (LOS), were found to be significantly lower when using the laparoscopic approach as compared with an open approach [49]. This meta-analysis included six mainly retrospective randomized studies with a lower mean age in laparoscopic groups, so the results may have some selection bias. A more recent meta-analysis (2019) yielded the same conclusions regarding postoperative mortality, complications, and LOS, also including lower complication rates following laparoscopy in the case of complicated appendicitis [50].

The rate of laparoscopies performed on the elderly varies significantly, ranging from 19.3% to 67% [12,31,46,51] depending on the year of the study (these are retrospective observational studies), but mainly depend on the preference of the surgeon.

There is a higher risk of conversion from laparoscopy to open appendectomy in elderly patients, reaching 3–17% [5,31]. The most common reason for conversion is periappendicular infiltration/an inflammatory mass.

After the successful conservative treatment of an appendiceal abscess or inflammatory mass, it is essential to plan interval appendectomy due to a higher probability of appendiceal tumors (5.9–20%) [5,52,53] after the age of 65 [54]. It is known that 28.6–58% appendiceal tumors present as acute appendicitis [55,56]. Interval appendectomy is safe to perform laparoscopically in selected individuals without co-morbidities [57].

Although extensive resections (ileocecal resection and right-sided hemicolectomy) are more common in elderly patients, increased age is not a significant predictor for extensive resections. Appendiceal mass, non-visualization of the appendix, delayed admission, and high levels of CRP are reported as preoperative factors for extensive resection [58]. Colectomy has been reported as the extent of surgery in 10.7–14.3% [12].

## 6. Morbidity and Mortality

Regarding acute appendicitis in all adult patients, there are several well-known risk factors for postoperative complications—increased age, open surgery, and complicated appendicitis [59]. With increasing age, there are more patients with comorbidities, although interestingly not all comorbidities or high American Society of Anaesthesiologists (ASA) scores (≥3) are associated with an increased rate of complications [5,60]. In the elderly, independent factors for postoperative complications include anemia, positive history of cardiac diseases, chronic renal insufficiency, and open access for the appendectomy [46,60]. Postoperative complications are also affected by the patient’s frailty. An increased frailty score is associated with an increased risk of postoperative complications in elderly patients undergoing emergency laparotomy [61]. Postoperative complications/overall morbidity in elderly patients range between 19.3–46.2% [5,31,46], as compared to 5.06–9.3% in younger patients [27,35]. The main cause of morbidity is related to surgical site infection, seen in 9.0–15.4% of cases; this frequency is higher than that in younger patients (2.6–3.7%) [5,31].

When comparing postoperative complications based on the Clavien–Dindo classification, there are different results regarding the impact of age on the grade of complications [59,60]. In a larger retrospective study, Moreira et al. concluded that age, open surgery, complicated appendicitis, ASA score (≥2), and increased surgery time affected the complication grade. Age above 80 also affects the complication grade and mortality [62]. Mortality ranges from 0.74% [46] to 6.1% [5] in the elderly and increases with age, while in the general population it is 0.04–0.21% [54,63,64]. Risk factors that are associated with higher mortality include increased frailty score, open surgery, and complicated appendicitis [9,49,61].

Increased morbidity is reflected in mean length of stay in hospital (LOS, 4.0–6.0 days) [12,31,39,62]. This increases to 7.8–11 days [12,62] in patients above the age of 80 as compared to 2.6–3.2 days [12,31] in the younger population. Some studies differentiate mean LOS in complicated appendicitis (6.5–8.3 days) [46,51] and uncomplicated appendicitis (3.2–3.56 days) [51].

## 7. Conclusions

This review shows that management of acute appendicitis in the elderly is not as straightforward as it is in the younger population. The elderly patient presents a diagnostic challenge regarding atypical presentations due to physiological changes with age, a wide variety of differential diagnoses, comorbidities and their associated polypharmacy, and an appropriate choice of imaging modality. Although we are constantly striving for evidence-based treatment, available data on elderly patients with acute appendicitis are mainly retrospective and based on smaller groups of patients. Thus, the treatment plan should be based on each individual case as per the institution or the surgeon’s preference. Even in elective surgery, complications are more pronounced in the elderly population, and these are even more prominent in acute settings such as that of acute appendicitis.

## Figures and Tables

**Table 1:** Comparison of different scoring systems (Alvarado score, RIPASA score, Lintula score).

	Alvarado Score [24]	RIPASA Score [25]	Lintula Score [26]
Sex	-	Female + 0.5	Female 0
		Male + 1	Male + 2
Age	-	<40 + 1	-
		>40 + 0.5	
Duration of symptoms	-	<48 h + 1 >48 h + 0.5	-
Intensity of pain	-	-	Mild/moderate 0 Severe + 2
Anorexia	1	1	-
Nausea or vomiting	1	1	2
Right iliac fossa (RIF) pain/tenderness	2	0.5 + 1 ^1^	4
Elevated temperature	1	2	3
Guarding	-	2	4
Rebound tenderness	1	1	7
Bowel sounds	-	-	Absent/tinkling + 4
			Normal 0
Rovsings sign	-	2	-
Migration of pain to RIF	1	0.5	4
Leukocytosis (>10,000)	2	1	-
Leukocyte left shift (>75%)	1	-	-
Normal urine analysis	-	1	-
Total	10	16	32
Points to rule out appendicitis ^2^	<3–5 (<3 [22])		≤15
Possible diagnosis of appendicitis	>6–7 (>6 [22])	>7.5	≥21

RIF: right iliac fossa. ^1^ Score combined: 0.5 points for right iliac fossa pain (symptom) and 1 point for right iliac fossa tenderness (sign). ^2^ In the general population. Score in brackets, possible points for the elderly.
